# Visible-Band Nanosecond Pulsed Laser Damage Thresholds of Silicon 2D Imaging Arrays

**DOI:** 10.3390/s22072526

**Published:** 2022-03-25

**Authors:** Christopher Westgate, David James

**Affiliations:** 1 Defence Science and Technology Laboratory, Porton Down, Salisbury SP4 0JQ, UK; 2 Defence Academy of the United Kingdom, Cranfield University, Swindon SN6 8LA, UK; d.james@cranfield.ac.uk

**Keywords:** pulsed laser, damage threshold, silicon camera, CCD, CMOS

## Abstract

Laser-induced camera damage thresholds were measured for several sensors of three different sensor architectures using a Q-switched Nd:YAG laser in order to determine their pulsed laser-induced damage thresholds. Charge coupled device (CCD), front-side illuminated complimentary metal-oxide semiconductor (FSI CMOS), and back-side illuminated (BSI) CMOS sensors were assessed under laboratory and outdoor environments by increasing the focused laser intensity onto the sensors and recording the sensor output. The damage sites were classified qualitatively into damage types, and pixel counting methods were applied to quantitatively plot damage scale against laser intensity. Probit-fits were applied to find the intensity values where a 95% probability of damage would occur (FD_95_) and showed that FD_95_ was approximately the same under laboratory conditions for CCD, FSI CMOS, and BSI CMOS sensors (mean 532 nm FD_95_ of 0.077 ± 0.01 Jcm^−2^). BSI CMOS sensors were the most robust to large-scale damage effects—BSI sensor kill was found at approximately 10^3^ Jcm^−2^, compared to 10 Jcm^−2^ for FSI CMOS, and between ~1.6 and 2.7 Jcm^−2^ for CCDs.

## 1. Introduction

**Figure 1:**
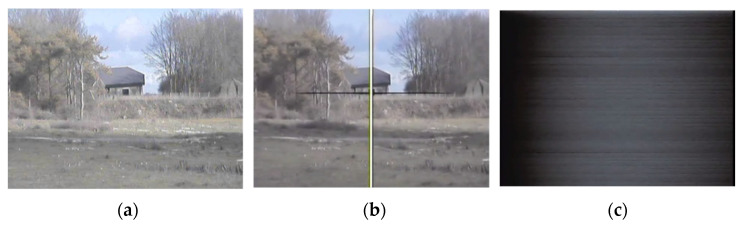
A visible-band camera images a building 500 m away: (**a**) shows the scene prior to engagement; (**b**) shows the first camera output effect after being illuminated by a pulsed laser system, causing irreversible line damage; and (**c**) shows full frame damage.

Pulsed laser technology, particularly Q-switched lasers that emit nanosecond-length pulses, are well-developed and prevalent on the modern battlefield. Pulsed lasers can be used for a multitude of roles, such as laser designations of targets for guided munitions and range-finding of distant objects. Pulsed lasers have also been implemented as countermeasures to electro optic systems, due to the high peak powers they are able to generate from their short pulse durations. Figure 1 shows laser damage to a visible-band CCD surveillance camera after exposure to pulsed laser light at a range of 500 m.

Sensors are particularly vulnerable to pulsed lasers because of optical gain, which can be described as the ratio of irradiance at the pixel to the irradiance at the objective lens and can be 10^6^ or more (e.g., gain ≈10^6^ for Ø_lens_ = 25.4 mm, *λ* = 532 nm and F# = 8). In simple terms, the large collection aperture of the lens system focusses the incoming laser pulse to a small, intense point on the electronic array. This intense laser focal spot can damage the electronic array by depositing thermal energy that is enough to break electrical connections, or fuse together connections that should be isolated. Furthermore, in-band laser wavelengths that are within the responsivity region of an imaging sensor are highly capable of causing laser-induced damage since the light-sensitive material in the sensor electronics is typically highly absorbing at these wavelengths.

The development of effective electro optic protection measures to protect sensors from laser damage requires knowledge of (a) the relevant threat lasers and their output parameters, and (b) the vulnerability of the sensors to be protected. In order to fulfil the knowledge gap in our understanding of sensor vulnerability to battlespace laser hazards and threats, their laser-induced damage thresholds should be measured. The measured thresholds can then be used to inform the protection requirements of future sensor systems—in the case of unknown threat wavelengths, the protection measures may be in the form of non-linear optical limiters.

Laser-induced damage thresholds (LIDTs) have been published in the literature over several decades by various groups. A few relevant studies of sensor damage are listed below:

1. Schwarz et al. have published several papers and proceedings on the LIDTs of camera sensors, most recently publishing the 527 nm picosecond thresholds of CCD and CMOS sensors at approximately 10 mJ/cm^2^ [1]. Previous work by Schwarz et al. found the 532 nm nanosecond thresholds of colour and monochrome CMOS and CCD sensors to range from 0.03 to 0.1 Jcm^−2^ [2,3,4].
2. Burgess et al. published the continuous-wave LIDTs of visible and shortwave infrared cameras using a 1070 nm source and laser exposure times from microsecond to continuous wave [5]. The objective plane irradiances to cause damage during a 0.5 ms exposure were 0.4 Wcm^−2^ and 0.7 Wcm^−2^ for silicon cameras (in focus) and SWIR sensors, respectively.
3. Becker et al. published LIDT data on CCDs using 1064 nm nanosecond pulses and found functional output changes from 0.55 Jcm^−2^ [6]. The article provides an interesting investigation into the damage morphology.
4. Guo et al. performed LIDT measurements on CMOS sensors using a 1064 nm pulsed laser and found the damage threshold to be 0.38 Jcm^−2^, with varying degrees of line damage starting between 0.64 and 1.0 Jcm^−2^ [7].

This study adds to previously-published data by sharing damage threshold data on a variety of different visible-band sensor architectures under laboratory and outdoor conditions. Outdoor testing was performed to observe any changes on sensor damage thresholds caused by atmospheric turbulence effects, which serve to distort the beam’s wavefront, which can affect how the beam focuses onto the detector.

## 2. Materials and Methods

Laser damage threshold experiments were performed on a range of imaging sensors, listed in Table 1. The sensors can be grouped into three different sensor architectures—charge-coupled device (CCD), front-side-illuminated (FSI) CMOS, and back-side-illuminated (BSI) CMOS.

CCD sensors use the photoelectric effect to convert photons into electrons at each pixel. CCDs utilise a single analogue-digital converter (ADC) and amplifier, and every pixel’s charge must be transferred to this circuitry to be converted into a digital value. In order for each pixel’s charge packet to reach the ADC, the charges across the entire array are shifted vertically down by one pixel by the vertical shift register. This causes the bottom row of charges to transfer into a horizontal shift register, which then shifts the charges one-by-one to the ADC, located at the end. Once the horizontal shift register is empty, the vertical register moves the charges down by one pixel, and the process repeats until all of the charges have been read [8]. Having a single ADC results in low electrical noise, but the shift registers require increased time and power to operate, compared with CMOS sensors.

CMOS sensors differ from CCDs by adding an amplifier to each pixel and utilizing row and column-select transistors to allow row-by-row readouts, also known as a ‘rolling shutter’ readouts. The random-access readouts allows for higher framerates at the expense of increased noise caused by the readout circuitry. More recently, digital pixel sensor (DPS) CMOS sensors have emerged, which employ ADC circuitry at every pixel in order to achieve massively parallel conversion and fast readout of the digital pixel values, thereby achieving a ‘global shutter’ [9].

**Figure 2:**
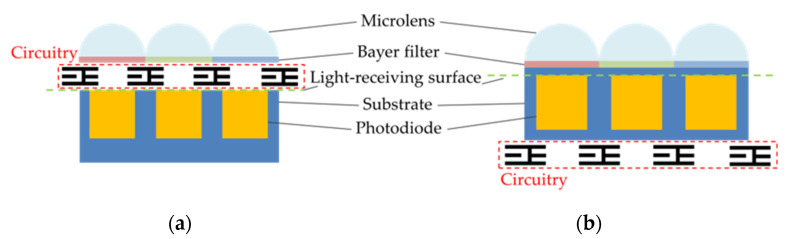
Schematic diagrams of: (**a**) a front-side illuminated sensor; (**b**) a back-side illuminated sensor.

CCD and CMOS sensors can both come in FSI and BSI variants, shown schematically in Figure 2. In an FSI sensor, the pixel electronics sit between the light-collecting microlenses and the photosensitive array. The pixel electronics block a proportion of the collected light, resulting in a ‘fill-factor’ determined by the ratio of photosensitive area to the circuit wiring area. The issue of fill-factor becomes more problematic as pixel sizes get smaller and smaller, because the wiring covers an increasing proportion of the pixel.

The BSI architecture was designed to overcome inefficiencies of the fill-factor problem for smaller pixel sizes. A BSI sensor is constructed by inverting the silicon wafer during manufacture and thinning the substrate, so that photons are able to reach the photodiode without passing through the circuitry layer, which would otherwise partially block incoming light.

The sensors were assessed under laboratory conditions, where a well-defined beam profile could be delivered to the device, and also under outdoor conditions where atmospheric turbulence affects the spatial beam profile. Both laboratory and outdoor experimental setups are described briefly in this section. More information on the techniques, considerations, and adaptations for the experiments performed in this study can be found within a short e-book by Westgate [10], which describes further adaptations and considerations for others performing these measurements.

The laser-induced damage threshold (LIDT) of the visible-band sensors were primarily measured at 532 nm. A small number of measurements were performed at 1064 nm; typically, visible-band sensors are not as vulnerable to near infrared pulses because many sensors employ infrared-cut filters in order to improve image contrast and quality.

### 2.1. Indoor Optical Setup

**Figure 3:**
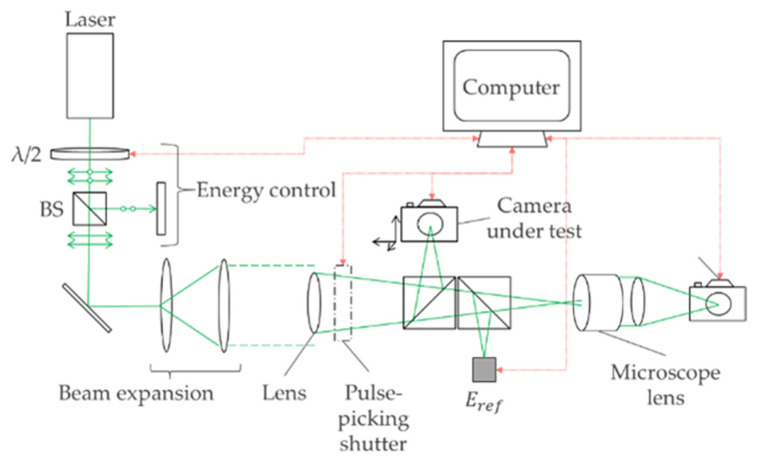
The laboratory optical setup for assessing the laser-induced damage threshold of cameras.

The laboratory optical setup is shown in Figure 3 and utilised a Quanta Ray GCR170 Nd:YAG laser (9 ns FWHM pulse width), frequency doubled to λ = 532 nm. A series of harmonic beamsplitters were used to pass only 532 nm pulses into the experiment—the 1064 nm pump wavelength was directed into a beam dump. The green pulses were passed through a *λ*/2 waveplate and polarizing beam splitter in order to provide energy control. The beam was then expanded to approximately 30 cm in diameter and re-collimated using a 5.08 cm diameter lens, to provide a more spatially uniform wavefront. (A large, expanded beam is more representative of a real-world engagement where a laser will have diverged to a size much larger than the objective lens, resulting in a plane wavefront). A 400 mm focal length Thorlabs singlet lens (p/n LA1725-A for 532 nm, p/n LA1725-C for 1064 nm) focused the spatially-uniform beam down to an Airy spot on the camera under test, which was mounted on an xyz-stage, via a first beam splitter. The effective F-number of the objective lens was $=\frac{f=400\;\mathrm{mm}}{d=50.8\;\mathrm{mm}}=7.9$. A second beam splitter directed a proportion of the beam to an Ophir PD10C calibrated reference energy meter. Finally, the focal plane was relay imaged onto a beam profiling camera through a Mitutoyo NIR 20x microscope lens, in order to measure the beam spot size with increased spatial resolution.

The measured focal spot sizes were taken to (a) determine the spot size at focus in order to calculate fluence, and (b) to check the spatial quality of the input beam and compare the beam spot size against theory. The $1/e^{2}$ spot sizes were used in this study to calculate fluence, and can be compared to the theoretical diffraction-limited $1/e^{2}$ spot diameter,$\;d_{0.135}$, which for an Airy disk is given by:

(1) $$d_{0.135}=1.64\times F\times \lambda$$

where *F* is the *F*-number and is equal to $\frac{focal\;length}{beam\;diameter}$ and λ is the laser wavelength. The measured spot sizes were $d_{0.135}=$ 12.36 µm at 532 nm, and $d_{0.135}=$ 18.82 µm at 1064 nm, which corresponded to spot sizes that covered just a few pixels on the cameras, depending on each camera’s pixel pitch. By contrast, the resultant spots on the beam profiling camera were ~20 to 30 px in diameter, because the magnifying optics of the beam profiler enabled finer observations of spot size and also focus position. The beam profiler was spatially calibrated at 0.68 µm/px.

It should be noted that the measured spot size at the beam profiling camera and at the camera-under-test can be different since both devices can be moved independently in the z-axis of the beam. Therefore, the fluences presented hereon are *estimated*, because small differences in focus position to the camera-under-test can result in slightly different values of spot size when compared to the measured spot size at the beam profiling camera. Atmospheric effects can also change the spot shape and size; Section 2.2 provides more details on this. The sensor fluence was defined in this study as:

(2) $$fluence_{sensor}=\frac{energy}{1/e^{2}\;spot\;area}=\frac{0.766\times \left(E_{ref}\times slope_{E}\right)\;}{\pi {\left(\frac{d_{0.135}}{2}\right)}^{2}}$$

where $slope_{E}$ is the calibration slope that determines the total energy to the camera-under-test from$\;E_{ref}$, the reference energy meter value. Moreover, 0.766 refers to the proportion of energy within the $1/e^{2}$ beam diameter (the diffraction-limited $1/e^{2}$ spot contains 76.6% of the total power of the beam).

Optimum focus on the camera-under-test was achieved when the peak intensity of the Airy disk was maximised by looking at the sensor output whilst moving the sensor in the z-axis. The laser was operated at low outputs and was attenuated using neutral density filters (in collimated-space) in order to avoid pixel saturation during the focus procedure. The background signal from the laser flashlamps was removed using a 532 nm notch filter in collimated-space, and all filters were removed before the test-runs began.

### 2.2. Outdoor Optical Setup

**Figure 4:**
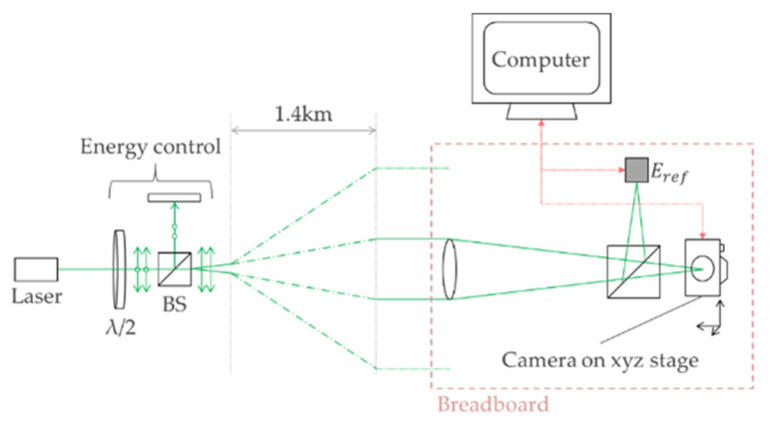
The optical setup for assessing camera damage thresholds outdoors.

The outdoor optical setup is shown in Figure 4. An Nd:YAG laser (Quantel by Lumibird, Lannion, France) with a 6 ns FWHM pulse width was passed through harmonic beam splitters, transmitting only green pulses to the target location, 1.4 km away. Laser energy control was achieved by adjusting the Q-switch timing delay, and further adjusted using a *λ*/2 waveplate and polarizing beam splitter. At the target location, the camera under test was positioned on an xyz translation stage and looked back at the laser building through the same 400 mm focal length Thorlabs lenses used in the laboratory tests. A beam splitter directed a proportion of the beam to a calibrated reference energy meter (Ophir PD10C).

The correct focus was achieved by moving the camera in the z-axis until the sharpest image of the laser-send building was achieved. Fine focus was then verified by sending a low-power CW alignment 532 nm beam to the camera from the send point and moving the camera to achieve the smallest spot size on the camera output. Though the singlet lens caused a degree of chromatic aberration to the scene image, it does not affect the spot size or shape for single-wavelength sources such as lasers.

Spot size measurements using the relay imaging arm were initially attempted in order to measure the changes in beam spatial profile caused by atmospheric turbulence. However, the slow framerate of the beam profiling camera (two frames per second) contributed to the difficulty in placing the beam profiling camera (BPC) at focus, which was compounded by focus-hopping in the z-axis caused by atmospheric scintillation. Future improvements to outdoor measurements will attempt spatial measurements of each pulse by changing the beam profiling camera.

For the purpose of this study, the measured spot sizes (under laboratory conditions) are assumed in the outdoor results. The limitation of using laboratory-measured spot sizes is that atmospheric turbulence primarily serves to reduce the focal-plane fluence when compared to a no-turbulence condition, by introducing wavefront aberrations that result in pulses that do not focus optimally [11]. Therefore the outdoor results presented in this paper overestimate the fluence at the sensor, since in reality the fluence is primarily reduced by the atmosphere and this reduction was not measured in this experimental setup.

### 2.3. Test Run Methodology (Indoor and Outdoor)

Once the camera-under-test had been placed at optimum focus of the laser, a one-on-one (a single exposure on a single site) test run was performed. The test started using low laser energies so that the sensor was not immediately damaged, and followed the steps below by Westgate [10] (text reproduced for clarity and relevance):

1. “A single pulse was fired at the sensor. For every pulse, the pulse number, reference energy meter reading, and damage effect (no effect, single pixel, multi-pixel, line, full frame) was recorded.
2. The output of the sensor was checked for an effect. The sensor was covered to check for bright pixel damage and illuminated uniformly using an external light source to check for dark pixel damage.
  - If no effect was observed, the energy was increased, and the sensor was translated diagonally so the next pulse would interact with fresh material. The sensor was re-focused if required.
  - If an effect was observed, the output of the sensor was saved, and the sensor was translated.
3. Steps one and two were repeated until all the required data was collected.”

Output images were saved with dark and uniformly illuminated backgrounds for later analysis. Shots at the ‘edge’ of a damage effect, for example, at the threshold between no damage and single pixel damage, were repeated in order to build up a statistical probability of damage in post-experiment analysis using data fitting tools—this is explained further in Section 2.4.1.

### 2.4. Data Analysis

Sensor damage effects are related more directly to focal-plane intensity as opposed to energy; therefore, the data in this study are presented in terms of focal-plane fluence (units Jcm^−2^). By presenting the focal-plane fluences (and the method by which the fluence is calculated), results can be compared between groups without needing to know details of the preceding optics or optical gain used in the experiment, as these parameters are already considered when arriving at the focal-plane fluence values.

The output images of the sensors were analysed, and the resultant effect of each pulse was classified into five levels as defined in Table 2 and used to create ‘damage effect vs. fluence’ plots. The damage effect plots are useful to identify where step changes in damage scale occur, for example between no damage and single pixel damage, or multiple pixel damage and line damage.

#### 2.4.1. Pixel Counting

A limitation of classifying damage into categories is that damage sites may vary largely in size but still fall under the same category of damage, for example ‘multi-pixel damage’ could mean a small damage cluster of 15 px, or a large cluster of thousands of px. Pixel counting was performed to present the level of damage more accurately within each effect. Pixel counting can also be combined with information about the sensor and lens system to estimate the field of view that would be obscured by a damage spot.

**Figure 5:**
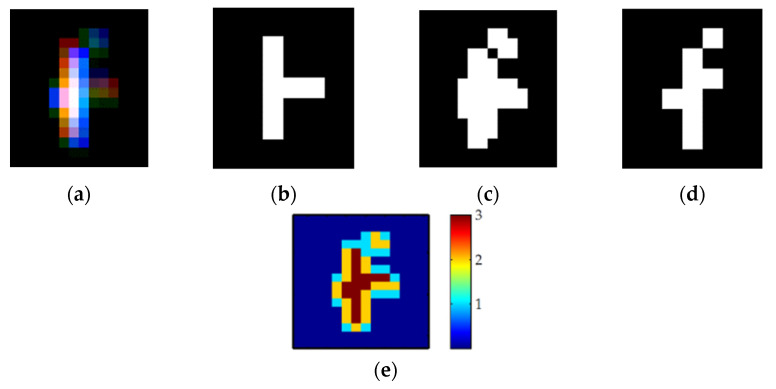
Image processing steps to determine the damaged pixel count: (**a**) laser damage site; (**b**) red channel pixels; (**c**) blue channel pixels; (**d**) green channel pixels; (**e**) superimposed pixel counts.

The output images of the damaged cameras were processed using a semi-automated pixel counting script using MathWorks MATLAB. The basic steps are shown in Figure 5 and are explained below for processing of images from a colour sensor that have bright pixel damage on a dark background:

1. A threshold value was calculated by multiplying the average background value by an arbitrary factor until correct discrimination of damage was manually observed. This threshold allows damaged pixels to be differentiated from the background and any temporal noise.
2. Each damage site was selected as a region of interest (ROI). The ROI was thresholded using the background threshold value to generate three binary images—one for each colour channel.
3. The total number of damaged pixels is calculated by superimposing all three binary images (from the red, green, and blue channels) together and counting the number of non-zero pixels.

For monochrome cameras there was only one output channel, resulting in a single binary image that was summed to find the total number of damaged pixels above the threshold level.

#### 2.4.2. Probit Fitting

A probit fit was used to generate fluence values (FD_x_) where the probability of damage equals x %. In order to run the probit model, the data were first compressed into a binary dataset whereby each pulse is categorised as a ‘damage’ or ‘no-damage’ event. For example, any pulses that resulted in damage, whether it was line damage or single pixel damage, would be categorised as a ‘1′. Occurrences of zero damage would be categorised as ‘0′. MathWorks MATLAB was then used to run the regression on the binary dataset to generate user-defined FD_x_ values.

**Figure 6:**
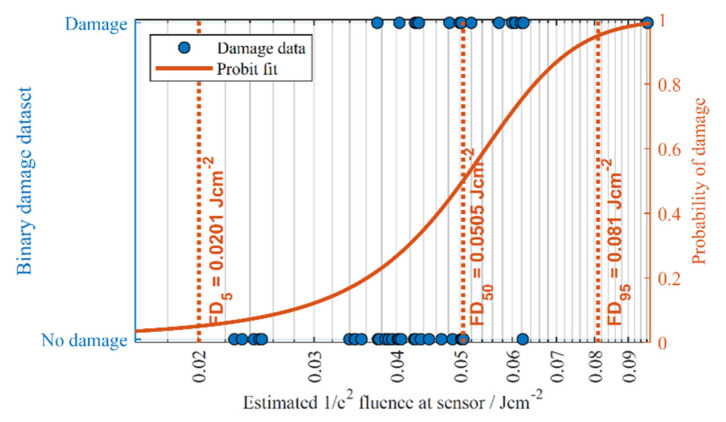
An example of a probit fit applied to CCD1 (colour) camera damage data.

An example of a probit plot is shown in Figure 6 for the colour CCD1 sensor assessed under laboratory conditions. The FD_95_ values generated by the probit fits for each sensor assessed in this study are summarized in the Results Summary section, alongside any sensor kill fluences.

## 3. Results

The results in this section are grouped by the three different sensor architectures that were tested—charge-coupled device (CCD), front-side illuminated (FSI) CMOS, and backside illuminated (BSI) CMOS. Pixel-counting plots and damage effect plots are presented.

All plotted results assume λ = 532 nm, unless specified otherwise. Data points measured in outdoor conditions are plotted with square markers with red outlines. 

### 3.1. CCD FSI

**Figure 7:**
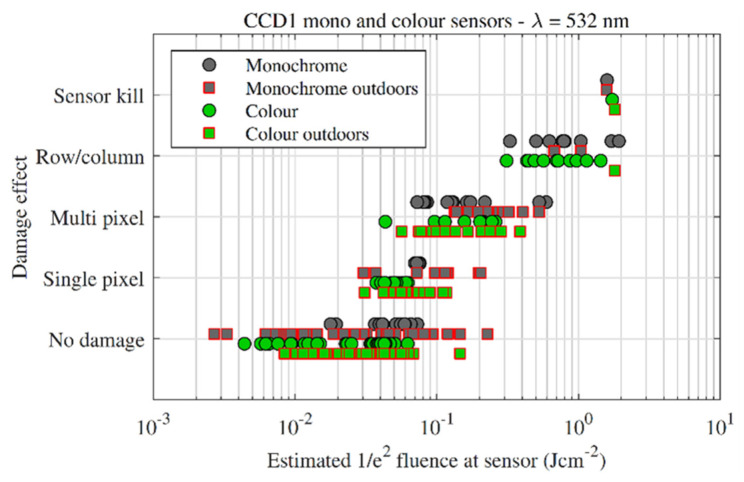
Damage effect vs. fluence for the CCD1 sensors. Indoor measurements are denoted by circles, outdoor measurements are denoted by squares.

Figure 7 plots damage effect vs. estimated focal-plane fluence for the monochrome and colour variants of CCD1. Under laboratory conditions (round markers), single pixel damage occurred at lower inputs for the colour camera compared with the monochrome camera. A similar difference between colour and monochrome cameras was also observed by Schwarz et al. [1] for CMOS sensors. Larger effects, such as multi-pixel damage, broadly required the same focal-plane fluences for both colour and monochrome variants, e.g., both variants transitioned from single-pixel damage to multi-pixel damage at around 0.1 Jcm^−2^. Similarly, the change from multi-pixel damage to line damage occurred roughly around 0.5 Jcm^−2^ for both variants.

The outdoor measurements followed the general trend of the lab measurements but with a larger spread of fluences that caused single pixel damage. The larger spread in fluences can be attributed to a change in ‘focusability’ of the pulses caused by atmospheric turbulence, i.e., the atmosphere affects the wavefront of the pulse causing the focal spot to change in shape. For most pulses this results in a reduction in fluence; however, occasionally, atmospheric turbulence can cause an increase in collected energy and, when combined with a high Strehl ratio pulse, can result in an *increase* in focal-plane fluence when compared with a no turbulence case [11]. This could explain the spread in outdoor fluences that are below the laboratory fluences that caused damage. In both outdoor and laboratory experiments, sensor kill was achieved between 1.58 and 1.80 Jcm^−2^.

**Figure 8:**
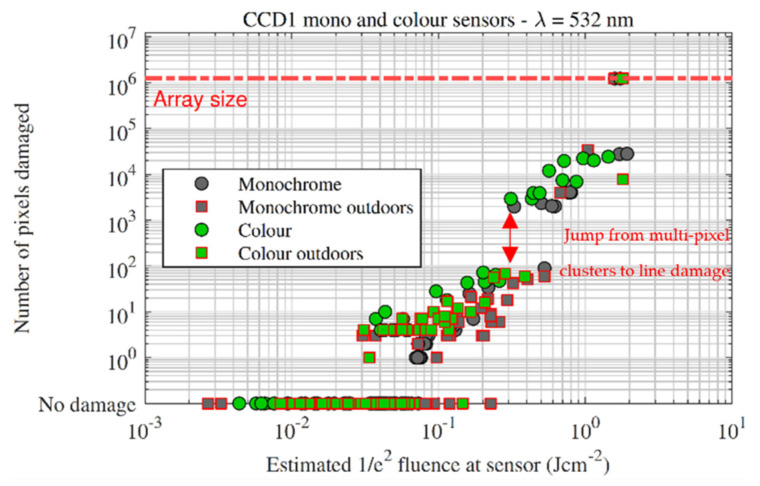
Damaged pixels vs. fluence for the CCD1 sensor. The sharp jump in pixel count indicated by the red arrow was caused by the transition from pixel cluster damage to line damage, where several columns were damaged at a time.

Figure 8 plots the pixel count vs. focal-plane fluence for the CCD1 sensor. The outdoor tests again show a wider spread of fluences for low numbers of damaged pixels, and the spread narrows as the damage scale increases. The jump in pixel count (hundreds of pixels to thousands of pixels), which was observed in all sensors (except for BSI) and correlates with the jump from multi-pixel clusters to line damage. The jump makes sense when one considers that (for the sensors in this study) even a single row or column consists of thousands of pixels.

**Figure 9:**
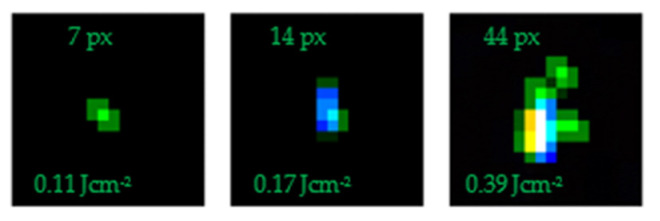
Examples of small-scale damage sites on the colour CCD1 sensor.

**Figure 10:**
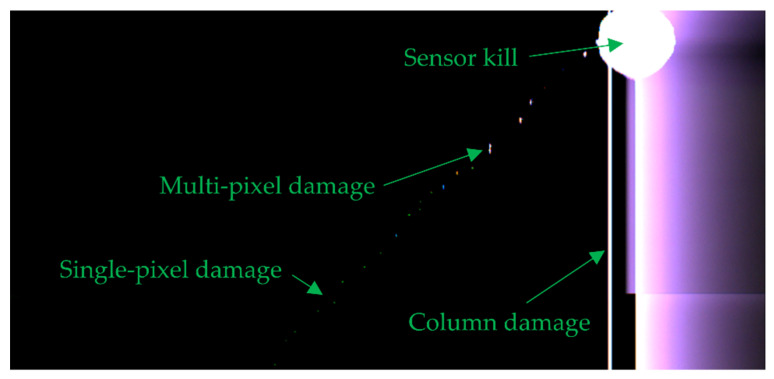
The output image of the colour CCD1 sensor after indoor testing where sensor kill was achieved. The image is taken as a bright light is pointed at the camera, showing no responsivity to the **left** side of the array, and a veiling signal to the **right**.

Figure 9 shows some examples of the small-scale damage caused at lower fluences on the colour CCD1 sensor camera, whilst Figure 10 shows a slightly cropped portion of the full sensor output at the end of the measurement run once sensor kill was achieved.

**Figure 11:**
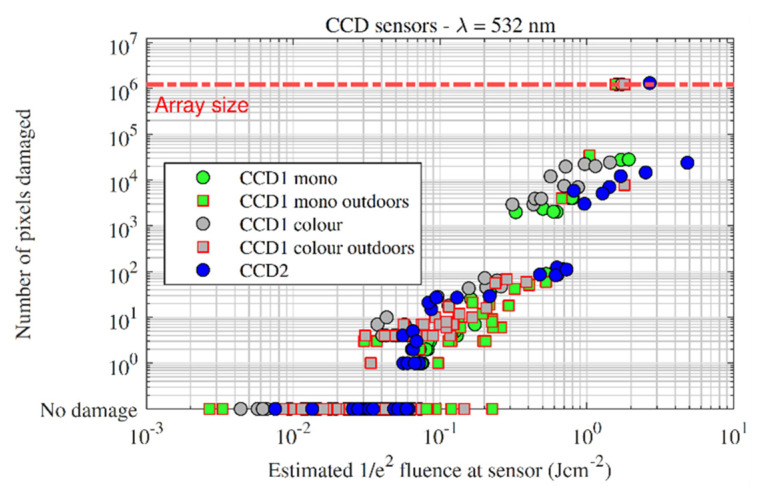
Damaged pixels vs. fluence for all of the CCD sensors measured in this study.

The CCD2 monochrome sensor was tested under laboratory conditions and is plotted over the CCD1 data in Figure 11. CCD2 followed a similar escalation of damage with fluence as the other CCDs, with sensor kill observed at 2.68 Jcm^−2^.

### 3.2. CMOS FSI

The damage thresholds of two FSI CMOS sensors were measured—CMOS1 was measured under laboratory conditions at 532 and 1064 nm, and CMOS2 was measured under outdoor conditions at 532 nm.

**Figure 12:**
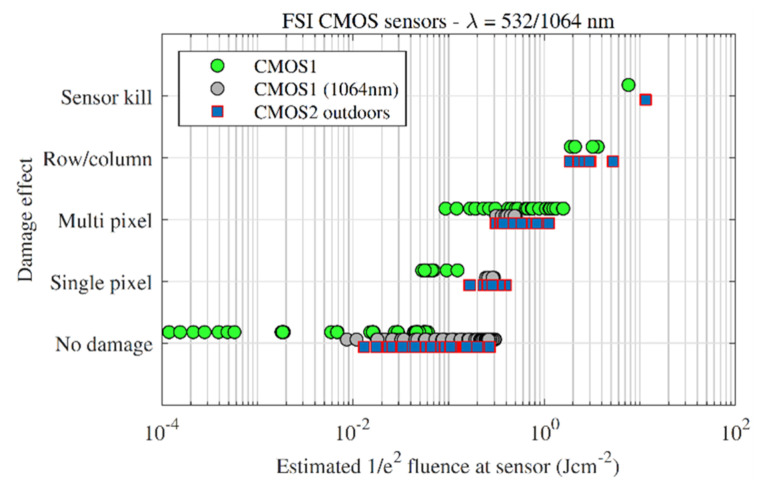
Damage effect vs. fluence for the front-side illuminated CMOS sensors. Indoor measurements are denoted by circles, outdoor measurements are denoted by squares.

Figure 12 presents the damage effect plot for the above tests. At 532 nm, the CMOS1 sensor escalated through single-, multi- and line-damage at approximately the same fluences as the CCD sensors in Section 3.1. Sensor-kill, however, was reached at a higher level of 7.6 Jcm^−2^, compared with an average of 2.0 Jcm^−2^ for the CCD under laboratory conditions.

**Figure 13:**
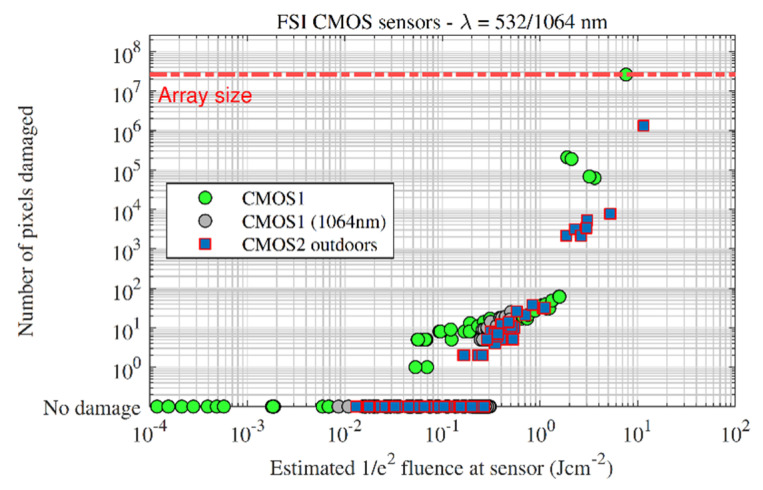
Damaged pixels vs. fluence for all of the front-side illuminated CMOS sensors measured in this study.

At 1064 nm, single pixel damage effects started at slightly higher fluences than at 532 nm; however, the number of data points are limited since this experiment was performed first and the array was preserved for the 532 nm measurements. Despite requiring more fluence at 1064 nm to start initiate single pixel damage, Figure 13 shows that the number of pixels damaged scaled similarly with fluence for 532 and 1064 nm, once the threshold for damage had been exceeded. The IR-cut filter was removed for the 1064 nm measurements.

The CMOS2 camera measurements were performed outdoors and follows the indoor data well, albeit ‘shifted’ to higher fluences. The shifting is caused by assuming all of the collected energy was focused into a diffraction limited spot, given the limitation of the experimental apparatus to measure the spatial profile of each pulse as described previously.

### 3.3. CMOS BSI

**Figure 14:**
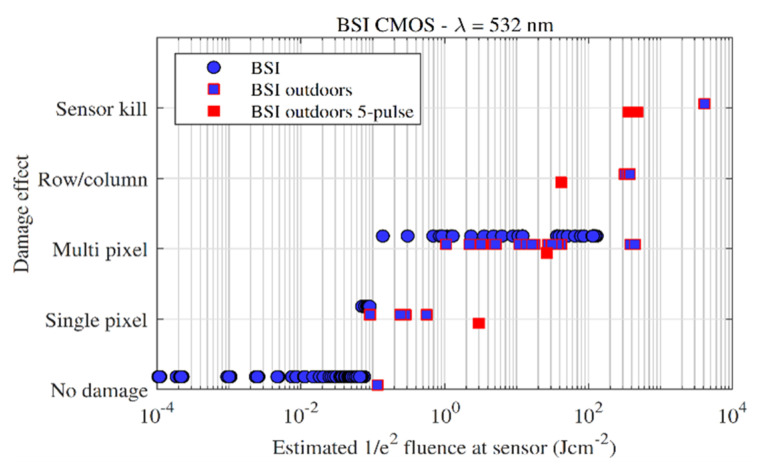
Damage effect vs. fluence for the back-side illuminated CMOS sensors. Indoor measurements are denoted by circles, outdoor measurements are denoted by squares.

**Figure 15:**
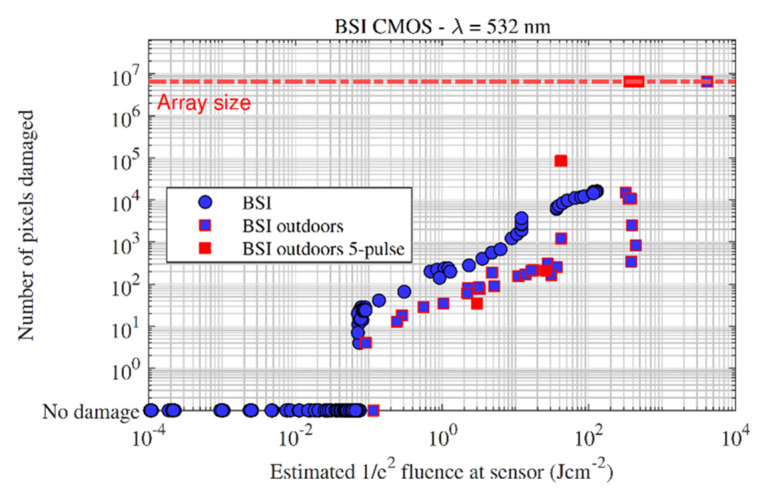
Damaged pixels vs. fluence for all of the back-side illuminated CMOS sensors measured in this study.

Indoor and outdoor measurements at 532 nm were performed on several BSI CMOS sensors (of the same sensor model); Figure 14 and Figure 15 show the damage effect plot and damaged pixels plot, respectively. The ‘5-pulse’ markers present the average estimated fluence of the five pulses. Both plots show that single-pixel and multi-pixel effects are generated at similar fluences compared comparable with the FSI CMOS and CCD sensors, i.e., single pixel damage occurred around the 0.1 Jcm^−2^ level, which then developed quickly to the multiple pixel damage level. For the laboratory experiment (round blue markers), the optical setup at the time limited the amount of energy that could be delivered to the sensor, so line damage and sensor kill could not be generated.

**Figure 16:**
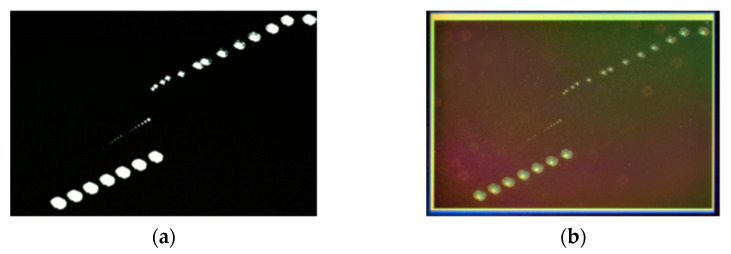
Post-test images of the BSI CMOS sensor. The sensor remained functional after these tests aside from the large cluster damage: (**a**) electronic damage (still image output); (**b**) low-magnification microscope image of the array showing physical damage to array.

The similarities fall away at higher fluences, where line damage and sensor kill on the BSI architecture required significantly higher fluences then the FSI CMOS (and CCD) to generate. The BSI CMOS sensors required thousands of Jcm^−2^ for single pulse sensor kill. Compared with the measured FSI sensors in Section 3.2, the BSI sensors here required approximately an order of magnitude increase in fluence to cause line damage, and two orders of magnitude to cause sensor kill. Figure 16 presents the sensor output image and the physical damage caused to the surface of the BSI sensor after laboratory testing, illustrating the high intensities that the sensor was able to survive. The increase in fluence required to cause large-scale effects in BSI sensors may be attributed to the location of the amplifier and readout circuitry, which is located physically below the photosensitive material and therefore offers a degree of protection.

The effect of multiple pulses was also investigated outdoors—at low input energies, five consecutive pulses (at the laser repetition rate of 10 Hz) did not change the scale of damage, i.e., single- or multiple-pixel cluster damage did not ‘grow’ significantly in size by increasing the number of pulses. This was observed qualitatively; at low inputs, the damage spots were simply ‘filled in’, with each successive pulse causing low-level damage within the focus spot. At high inputs, however, the level of damage increased dramatically with each successive pulse, possibly because each pulse makes a stronger contribution to drilling and ablation of the array. Thus, sensor kill was achieved at lower fluences (approximately 10^2^ instead of 10^3^ Jcm^−2^) for a five-pulse event when compared to a single pulse.

### 3.4. Results Summary

The collective FD_95_ and sensor kill values are presented in Table 3. The sensor kill values are indicative of the order of magnitude required, as opposed to the absolute threshold to cause sensor kill—this is because the damage sites become increasingly larger as the laser energy approaches sensor kill and quickly reduces the amount of usable array area with which to interact with. Thus, energies are sometimes ramped up quickly near sensor kill, and may ‘overtake’ the actual kill threshold.

**Figure 17:**
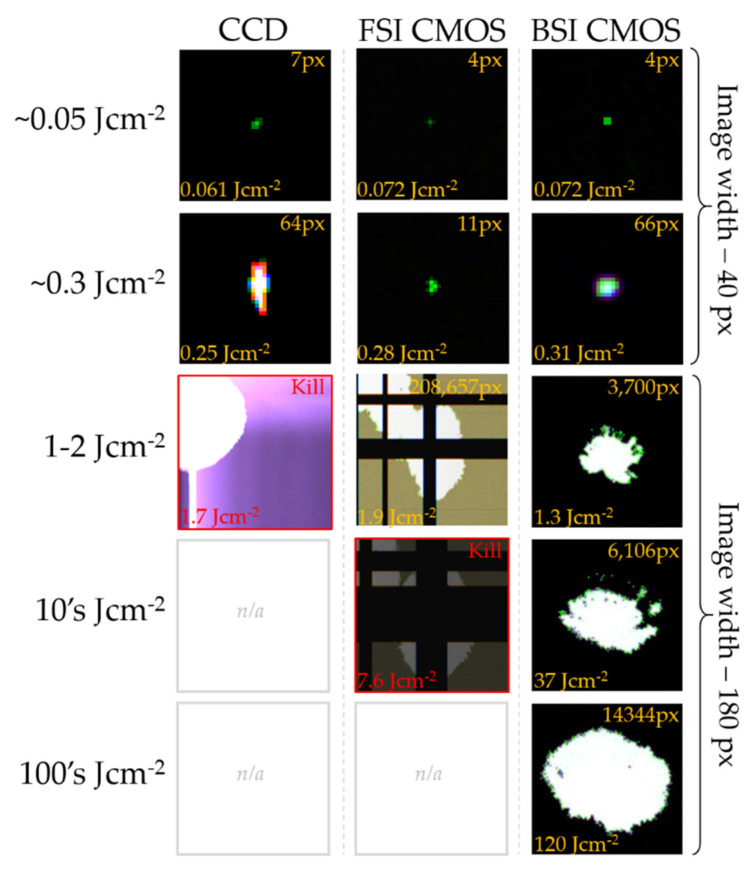
Damage site images from the indoor measurements of CCD1, CMOS1, and BSI sensors at 532 nm.

Figure 17 presents a selection of damage sites generated on sensors from the three architectures tested under laboratory conditions. For fluences between 0.05 and 0.3 Jcm^−2^, single-pixel and multi-pixel clusters can be expected for all three architectures. For the CCD tested, fluences slightly above 0.3 Jcm^−2^ quickly resulted in line damage, and sensor kill was achieved between 1.6 and 1.8 Jcm^−2^. At the CCD sensor kill level, the FSI CMOS sensors remained functional but repeatedly exhibited line damage, with sensor kill occurring in the tens of Jcm^−2^. For the FSI CMOS in Figure 17, sensor kill was declared after a laser pulse resulted in a global darkening of the image. The BSI CMOS sensors remained functional at hundreds Jcm^−2^, with medium-sized cluster damage.

Resultant white pixel damage was commonly observed at higher inputs, where the laser intensity was high enough to damage the pixels that correspond to red, green and blue outputs under the colour filter array. At 532 nm, green pixel damage was most common for low inputs that resulted in single- and small multi-pixel damage sites because the colour filter array for the green pixels has the greatest transmission to the laser wavelength. In these cases of green-only pixel damage (and when the spot size covers more than a single green pixel), the colour filter array is expected to have provided enough linear transmission loss to the red and blue pixels to protect them from damage. Green pixel damage was also observed around the periphery of large damage sites. Occasionally, other resultant pixel colours were observed where various combinations of red, green, and blue pixels were damaged.

## 4. Discussion

The nanosecond damage thresholds of several imaging sensors were measured under laboratory and outdoor environments using a Q-switched Nd:YAG laser. The main findings of this study are:

- The FD_95_, or fluence to cause a 95% chance of damage, for CCD, FSI CMOS, and BSI CMOS sensors were all approximately the same under laboratory conditions, resulting in a mean 532 nm FD_95_ of 0.077 ± 0.01 Jcm^−2^.
  ○ For outdoor test results, the FD_95_ is skewed high and varies with the level of atmospheric turbulence, because the beam is assumed to focus to a diffraction-limited spot for these measurements; in reality, the fluence at the sensor would be reduced by atmosphere turbulence (for most pulses).
- Large-scale damage effects, such as line damage, were reached at lower fluences for CCD sensors compared with CMOS and BSI CMOS sensors. This may be related to the shift-register readout circuitry of the CCD sensors.
- The BSI CMOS sensor-kill level is approximately two orders of magnitude greater than the FSI CMOS sensors tested (10^3^ vs. 10 Jcm^−2^, respectively), and is likely the result of the pixel circuitry location being placed behind the photosensitive area, providing inherently protected electronics.

In order to protect against permanent sensor damage caused by pulsed lasers, laser protection filters can be employed. However, for a laser threat with unknown wavelength, simple fixed filters cannot be used since knowledge of the wavelength is a pre-requisite to their design. Therefore, a suitable protection filter must operate over a wide range of wavelengths, and it must also activate and block the incoming pulse on a timescale shorter than that of the actual pulse itself. The solution to such a problem lies with optical limiters, which are self-activated, intensity-based filters that attenuate high-intensity light but allow low-intensity light to pass through.

Optical limiters have been actively researched and published since the 1980s. Review papers covering decades of optical limiter research can be found online [12,13], and most recently by Gadhwal [14]. However, only limited published results of optical limiters actually demonstrated for sensor protection can also be found online because this is a topic area that could be considered sensitive. Dental et al. demonstrated a soluble tin naphthalocyanine reverse saturable absorber and presented results showing the limiter providing some degree of protection to a CCD [15].

An ideal optical limiter for visible-band sensor protection would limit the intensity to a level below the single pixel damage threshold for the sensor of interest. The optical limiter would also have high linear transmission in the ‘pass’ state, good optical clarity, exhibit zero coloration, be robust to the environment (for example against heat cycling) and not degrade over time, and be simple to manufacture and integrate onto a sensor.

## Figures and Tables

**Table 1:** Details of all the camera devices assessed in this study. Pixel size refers to the length of one edge—pixels in all the sensors tested within are square. * FSI = front-side illuminated, BSI = back-side illuminated.

Name	Mono or Colour	Shutter	Type *	Px Size/μm	Res/px
CCD1	Mono + colour	Global	CCD FSI	3.75	1280 × 960
CCD2	Mono	Global	CCD FSI	6.45	1280 × 1024
CMOS1	Colour	Global	CMOS FSI	4.5	5120 × 5120
CMOS2	Mono	Global	CMOS FSI	5.3	1280 × 1024
BSI	Colour	Global	CMOS BSI	2.4	3088 × 2076

**Table 2:** Damage effect definitions.

Damage Effect	Definition
No damage	Camera output unaffected after the laser event.
Single-pixel	Permanent damage to a single pixel or group of pixels smaller than the laser spot size.
Multi-pixel	Permanent pixel damage of a cluster of pixels greater than the laser spot size.
Line	Permanent pixel damage that results in row/column effects.
Sensor kill	A global effect is caused to the sensor, e.g., the sensor output could fail completely, or the whole-array responsivity could be affected.

**Table 3:** Summary table of the assessed sensors and their associated FD_95_ values. Kill levels are simply where kill-level effects were first observed and are not necessarily the lowest fluence at which kill can be achieved. † Five-pulse event.

				Estimated 1/*e*^2^ Fluence (Jcm^−2^)	
Name	Type	Setting	*λ*/nm	FD_95_	Kill
CCD1 (mono)	CCD FSI	Lab	532	0.075	1.59
		Outdoor	532	0.23	1.58
CCD1 (colour)	CCD FSI	Lab	532	0.081	1.73
		Outdoor	532	0.11	1.80
CCD2	CCD FSI	Lab	532	0.067	2.68
CMOS1	CMOS FSI	Lab	532	0.069	7.61
		Lab	1064	0.34	Not tested
CMOS2	CMOS FSI	Outdoor	532	0.35	11.5
BSI	CMOS BSI	Lab	532	0.091	>129
		Outdoor	532	0.22	4110, 358 †

## Data Availability

The data generated from the experiments described within this paper are not publicly available at this time but may be obtained from the authors on a case-by-case basis.

## Disclaimer

© Crown copyright (2022): Dstl. This material is licensed under the terms of the Open Government Licence except where otherwise stated. To view this licence, visit http://www.nationalarchives.gov.uk/doc/opengovernment-licence/version/3 or write to the Information Policy Team, The National Archives, Kew, London TW9 4DU, or email: psi@nationalarchives.gov.uk.
